# Evaluation of Nano‐curcumin effects against Tartrazine‐induced abnormalities in liver and kidney histology and other biochemical parameters

**DOI:** 10.1002/fsn3.2790

**Published:** 2022-03-21

**Authors:** Gaber E. El‐Desoky, Saikh M. Wabaidur, Zeid A. AlOthman, Mohamed A. Habila

**Affiliations:** ^1^ 37850 Department of Chemistry College of Science King Saud University Riyadh Kingdom of Saudi Arabia

**Keywords:** curcumin, experimental rats, nanomaterials, tartrazine, therapeutic effects

## Abstract

In the current study, 40 albino male rats were investigated to evaluate the impact of Nano‐curcumin (Nano‐CUR) administration against Tartrazine (TZ)‐induced variations in kidney and liver histology and their related functions. The liver function biomarkers are (glutamate oxaloacetate transaminase (GOT), glutamate pyruvate transaminase (GPT), alanine aminotransferase (ALT), aspartate aminotransferase (AST), gamma‐glutamyl transaminase (GGT), alkaline phosphatase (ALP), total bilirubin (T. BiLL)), whereas the kidney biomarkers are (creatinine, uric acid, urea, globulin, total protein (TP)), as well as blood parameters of (serum glucose (sGlu), alpha‐fetoprotein (AFP), protein Kinase‐C (PKC)) and lipid profiles that include (total lipids (TL), triglyceride (TG), total cholesterol (TC), low‐density lipoprotein‐cholesterol (LDL‐C), high‐density L‐C (HDL‐C), and very‐low‐density L‐C (VLDL‐C)). The collected rats were randomly separated into four different groups (G1, G2, G3, and G4) of 10 rats each, where G1 stands for control, G2 for TZ‐ingestion, G3 for Nano‐CUR‐ingestion, and G4 for (TZ + Nano‐CUR mix.) ingestion. TZ‐ingestion significantly (*p* < .05) increases the liver function enzymes’ activity, total bilirubin and kidney biomarkers (creatinine, urea, uric acid, total protein (TP), globulin (Glu)). Also, TZ‐ingestion significantly increased sGlu, PKC, AFP, as well as lipid profiles, while there were significant (*p* < .05) decreases in HDL‐C and albumin (Alb) concentrations compared to control. Histopathological changes in liver, such as dilatation of blood sinusoids and central vein with hemorrhage and necrosis, were observed due to TZ‐ingestion. Similarly, TZ‐ingestion influenced kidney tissues in terms of tubular dilatation with tubular degeneration, thickened basement membrane, and dilatation of the glomerular capillaries. Markedly, the administration of Nano‐CUR significantly decreased liver and kidney function enzymes as well as sGlu, AFP, and PKC, whereas it significantly increased serum Alb and HDL‐C levels compared to control and TZ‐ingested rats. All values arranged around normal control values. Also, the liver tissue of Nano‐CUR‐ingested rats showed a normal arrangement of normal blood sinusoids(s), hepatic cords, and hepatocytes as compared to controls. The same results were also found in the section of rat kidney ingested with 2.00 g of Nano‐CUR/(kg B.W.) showing near‐normal architecture as compared to control rats. The liver tissue of rats ingested by a mixture of (7.5 mg of TZ + 2.0 g of Nano‐CUR/kg B.W.) showed little necrosis. Similarly, a section of rat kidney ingested a mixture of (7.5 mg of TZ + 2.00 g of Nano‐CUR/kg B.W.) which revealed mild tubular degeneration and dilatation of the glomerular capillaries. These results support the protective and therapeutic effects of Nano‐CUR on the histology of liver and kidneys and their related function biomarkers. Also, Nano‐CUR corrects the imbalance in serum glucose (sGlu), AFP, PKC, and lipid profiles in TZ‐ingested rats compared to control.

## Graphical abstract

Albino male rats were investigated to evaluate the impact of Nano‐curcumin (Nano‐CUR) administration against Tartrazine (TZ)‐induced variations in kidney and liver histology and their related functions. The results support the protective and therapeutic effects of Nano‐CUR on the histology of the liver and kidneys and their related functions. Also, Nano‐CUR corrects the imbalance in serum glucose (sGlu), alpha‐fetoprotein (AFP), protein Kinase‐C (PKC), and lipid profiles in TZ‐ingested rats compared to control.

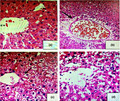

## 1 INTRODUCTION

**FIGURE 1:**
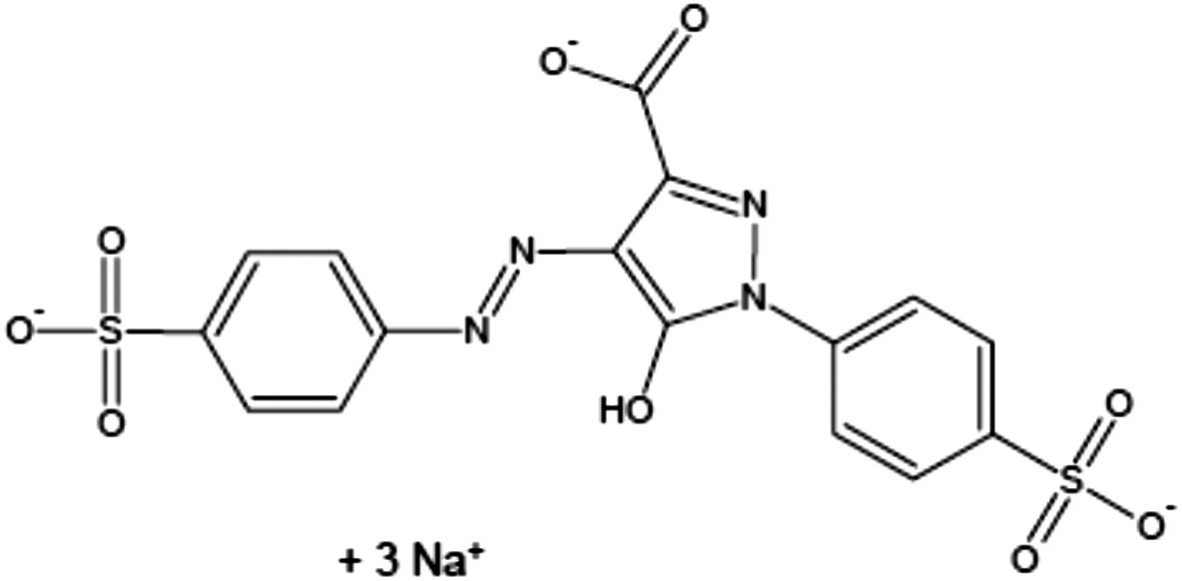
Chemical structure of Tartrazine

People consume various food additives every day, which have both advantages and disadvantages. Food additives have showed an important impact on food sources and provided people delicious, nutritious, and safe foods (Himri et al., 2011). Food additives are classified into six main groups including preservatives, nutritive additives, flavoring, coloring, texturizing, and miscellaneous compounds (Amin & Al‐Shehri, 2018). Food colors are added to give a color to a foodstuff and to restore its usual color, which is usually attractive to consumers, especially children. The synthetic food colors are considered as the class of essential food additives for many industries. Among the food colors, which are widely used, is Tartrazine (TZ), an orange‐colored water‐soluble powder, Figure 1. The consumption of food colored with TZ is increased in children without any control and may be aggravated in an adult as well, and in studies from Asia and Africa, exposure to synthetic colors like TZ and sunset yellow surpassed the acceptable daily intake (ADI), especially during festive and wedding times (Rao & Sudershan, 2008). Several foods with TZ in different quantities are soft drinks, sweetmeat, cereals, cotton candy, sauces, chips, soups, cake, jam, chomping gum, ice cream, marmalade, noodles, yogurt, and several expediency foods. Also, TZ was found in cosmetics, soaps, shampoos, and hair products. The medicinal preparations containing TZ are vitamins, antacids, and medical capsules (Walton et al., 1999). Young people consume such colors daily in the form of gum, chocolates, drinks, and chips.

Accruing research has reported the adverse effects of TZ on health, such as allergy, asthma, mutagenesis, and carcinogenesis (Chung et al., 1992; Moutinho et al., 2007). The intestinal or hepatic azo reductase in mammals reduces the azo‐dyes to form aromatic amines and N‐hydroxyl derivatives (Chequer et al., 2011; Demirkol et al., 2012). This mechanism is responsible for various disorders in human beings including pathological lesions in the brain, liver, spleen, and kidney. In addition, TZ revealed a significant rise in renal and liver function markers (Amin et al., 2010). In a recent study, it was reported that TZ induced a sharp deficiency in the biomarkers of antioxidants and a marked rise in MDA levels (El‐Desoky et al., 2020a). The TZ dyes’ exposure dislocates the equilibrium between the antioxidant and pro‐oxidant system and derives unnecessary reactive oxygen species (ROS), and results in cell apoptosis and DNA or protein damage (El‐Desoky et al., 2020a). Moreover, nervous system, behavior, and other metabolic and organ functions’ disorders are more easily affected in young people than adults.

Considering the hazardous effects of the azo‐dye, great attention has been given to its applications, by replacing or mixing it with a natural coloring material like annatto, curcumin, malt color, or beta‐carotene (El‐Desoky et al., 2020a), to prevent or reduce its bad effects.

Curcumin (CUR) is a polyphenol compound that is obtained from rhizome of *Curcuma longa* (turmeric) and is found to be safe at a dose of 8 g/kg/d (Siviero et al., 2015). Curcumin has shown its applications as an anti‐inflammatory, antioxidant, hypoglycemic, anticancer (Siviero et al., 2015), and anti‐Alzheimer agent (Wang et al., 2016). Also, curcumin has been used in pharmacological applications and as therapeutic drugs (Boarescu et al., 2019; Bulboacă et al., 2019). The usage of CUR with or without synthetic colors in coloring foodstuffs protects the foods from oxidative damage by strengthening the cell antioxidants. However, the extremely low solubility of CUR in water media (0.6 mg/ml in pure water) and very low oral bioavailability limit the applications of CUR (Kuri et al., 2017). Many studies have focused on improving its solubility in water via mechanical techniques and ultrasonic homogenization to make smaller solid dispersion (Chakraborty & Boolchand, 2014). The synthesis of Nano‐curcumin (Nano‐CUR) increased its solubility in water, widespread usage with or without synthetic colors, enhanced oral bioavailability (Wang et al., 2016), governing the oxidative stress, and provided protection to living cells against the ROS‐mediated oxidative damage (El‐Desoky et al., 2020a).

In the present study, Nano‐CUR was prepared and applied in combination with TZ that might be used for coloring drug, foodstuffs, and cosmetics. Additionally, it is used to evaluate the effects of TZ, Nano‐CUR, and a mixture of TZ and Nano‐CUR ingested to rats on blood parameters, kidney and liver function biomarkers, total bilirubin, and histological changes in liver and kidney tissues. The obtained results indicated that the proposed method can be successfully applied for the evaluation of Nano‐curcumin (Nano‐Cur) effects against azo‐dye‐induced disorders in blood parameters as well as damage of liver and kidney histology.

## 2 MATERIALS AND METHODS

### 2.1 Chemicals

Tartrazine (C.I. 19140, CAS No. 1934‐21‐0, MW 534.37) was acquired from Sigma‐Aldrich (St. Louis, MO, USA). The manufacturer assured a purity of 86.7%. Curcumin (CUR) was also procured from Sigma‐Aldrich Chemical Company. All other chemicals used were of analytical reagent grade. Deionized (DI) water was used throughout the experiments.

### 2.2 Nano‐CUR preparation

The method described by Aditya et al., (2017); Gopal & Gupta, 2016) was followed for preparation of the Nano‐CUR, in which a fixed quantity of CUR was mixed with a water/ethanol mixture and kept for 30 min under ultrasonic waves. The obtained mixture was then heated at 60°C for 2 h, resulting in the development of a suspension of CUR‐nanoparticles. The CUR‐nanoparticles were then separated using centrifugation and stored under freeze‐drying until their further uses. Transmission electron microscopy (TEM) technique was used to determine the shape and particle size of the prepared Nano‐CUR. On the other hand, the surface morphology of the produced Nano‐CUR was characterized using scanning electron microscope (SEM) of model S‐3400‐*N* (Hitachi, Japan). A particle size analyzer instrument Coulter Model LS 130 (Indianapolis, IN, USA) was used to determine the particle distribution. The prepared samples were suspended in DI water followed by sonication for 1 min with 500 W, Vibra Cell (Newtown, CT, USA) ultrasound system and added into a transparent cell. The crystalline pattern of the synthesized materials was investigated with an X‐ray diffraction analyzer, D8 FABLINE, Buker (Newtown, CT, USA). The related results can be found in our previously published research paper (El‐Desoky et al., 2020b).

### 2.3 Animals

Wister rats (male albino category) of weights in the range of 200–250 g were procured from the King Saud University Animal House (Riyadh, Kingdom of Saudi Arabia). Before the start of the experiments, they were kept in polypropylene cages for 7 days to adapt to the laboratory conditions, of photoperiod 12–12 h light–dark cycles, temperature 23 ± 1°C, and a humidity of 50 ± 15%. The rats were fed a standard basal diet with compositions of 5% fat, 65% carbohydrate, 20.3% protein, 5% fiber, 3.7% salt mixture, and 1% of vitamins and water ad libitum. The current experimental work was completed in the Pharmacy College animal house (King Saud University) following the guidelines of the Animal Ethics Committee of the Pharmacy College, King Saud University, KSU‐REC 008E.

### 2.4 Experimental design

Four different groups (G1, G2, G3, and G4) containing 10 rats each were separated using polypropylene cages, where the term G1 stands for orally ingested rats with 1 ml of DI water every day for continuous 50 days and considered as the control group; the orally ingested rats with 7.5 mg/kg B.W. TZ (prepared in 1 ml of DI water) each day for a 50‐day span were denoted as G2; the rats that orally swallowed Nano‐CUR 2.0 g/kg B.W. (prepared in 1 ml of DI water) regularly for 50 days were categorized as G3; and the mixture of 2.0 g Nano‐CUR + 7.5 mg of TZ/kg B.W. dissolved in 1 ml of DI water, orally ingested to rats daily for 50 days, were considered as G4. The colors (TZ and Nano‐CUR) were introduced to the nonfasted rats during the time interval between 9 and 10 a.m.

### 2.5 Collection of samples

Blood samples from the tested rats of each group were taken out with a glass capillary from the orbital sinus of fasting rats, as described by Hassan et al., (2014). All the blood samples were then relocated into glass centrifuge tubes (nonheparinized) and kept at room temperature until clotting followed by centrifuged for 15 min at a speed of 3500 × *g*. Blood serum was utilized for the measurement of liver (AST, ALT, GGT, ALP, T. BiLL), TP, Alb, Glu, Alb/Glu), kidney functions (creatinine, urea, uric acid), lipid profile (TG, TC, LDL‐C, HDL‐C, and VLDL), glucose levels of alpha‐fetoprotein, and protein Kinase‐C using commercially available assay kits. Mild anesthesia using diethyl ether was given to the rats for taking out the liver and kidneys of the rats. The evacuated body parts were washed out using cold saline buffer and sent for histological investigation. The relative masses of specific livers and kidneys were measured on the day of sacrifice of the rats.

### 2.6 Histopathological analysis

A decapitated part of the liver and kidney was stored in 10% neutral buffered formalin for the histopathological experiment and was additionally processed using the standard methodology. The microsections of the organ of 5‐μm thicknesses were stained with hematoxylin–eosin (H&E) before their histopathological examination under a light microscope. Fixed tissues were processed routinely, embedded in paraffin wax, sectioned into 5‐μm thick sections in a rotary microtome, and then stained with the H&E dye. At least three different sections were examined per sample of organ. The pathologist evaluating liver or kidney sections was unaware of the treatment the rat had received.

### 2.7 Biochemical parameters

#### 2.7.1 Biochemical parameters of hepatic marker enzymes

Serum AST and ALT activities were estimated according to the kinetic methodology defined by Schumann et al., while the activity of serum ALP was determined using commercial kits according to the EL‐Aaser and Merzabani methods, respectively (El‐Aaser & El‐Merzabani, 1975; Schumann et al., 2002). An efficient colorimetric method was used for the determination of serum gamma‐glutamyl transferase (GGT), as described by Fuke et al., (1976). The AST, ALT, GGT, and ALP activities are expressed as units per liter (U/L) throughout the manuscript. Serum total bilirubin was assessed by following the colorimetric method of Walter & Gerade, (1970).

#### 2.7.2 Determination of glucose, alpha‐fetoprotein, and protein Kinase‐C

Fasting blood glucose level, protein kinase‐C, and alpha‐fetoprotein were estimated according to the reported methods (Barham & Trinder, 1972; Bates, 1991; Wilkinson & Hallam, 1994; Wu et al., 1988).

#### 2.7.3 Lipid profile

The serum HDL was calculated following the Burstein et al. method (Burstein et al., 1970). The concentration of serum LDL‐C was assessed, according to the Wieland and Seidel colorimetric method (Wieland & Seidel, 1983). Serum total cholesterol and lipids’ concentrations were determined by applying the Richmond as well as Zollner and Kirsch methods, respectively (Richmond, 1973; Zollner & Kirsch, 1962). While the serum triglycerides were measured following the Trinder enzymatic colorimetric method (Trinder, 1969).

#### 2.7.4 Kidney functions

A simplified enzymatic/colorimetric method of Tabacco was adopted for the estimation of serum urea concentration (Tabacco et al., 1979). Serum creatinine and total protein were determined according to the methods of Heinegard and Tiderstrom and Krohn, respectively (Heinegård & Tiderström, 1973; Krohn, 2011). Uric acid was measured according to the method of Barham T (Barham & Trinder, 1972). Serum Alb concentration was determined based on the method of Doumas et al., while the Glu was determined by subtracting the Alb quantity from the total proteins (Doumas et al., 1971).

#### 2.7.5 Statistical analysis

All the obtained results for various groups and controls were expressed as means ± SE. The analysis of variance (one‐way ANOVA) with the statistical package for Social Sciences (SPSS) for Windows Version 10.0 was used for determining the significant differences among values. Differences were considered significant at *p* ≤ .05 level of significance.

## 3 RESULTS

### 3.1 The influence of TZ‐ and Nano‐CUR‐ingestion on liver and kidney weights

No death was recorded in all experimental animals, even after 50 days of the experiment. TZ treatment (G2) caused a significant (*p* < .01) reduction in body weight compared to the control group (G1). There was a significant increase in relative liver and relative kidney weights in G2 rats compared to G1 rats (Table 1). The administration of a mixture of Nano‐CUR and TZ to G4 rats showed substantial increases in body weight gain, while a significant decrease in relative liver weight and kidney weights was observed compared to G2 rats (Table 1). The incorporation of only Nano‐CUR into the G3 rats showed a significant (*p* < .05) increase in body weight and body weight gains, whereas a significant decrease in relative liver and kidney weights was noted compared to G2 rats. No apparent changes were detected between G1 and Nano‐CUR groups (G3 and G4) for the body weight, body weight gain, relative kidney and liver weights (Table 1). These results propose that the ingestion of Nano‐CUR can improve the conditions of body weight, relative liver and relative kidney weights during pretreatment.

**TABLE 1:** Effect of Tartrazine (TZ) and Nano‐curcumin (Nano‐CUR) on different parts in male albino rats

Groups→ Parameters↓	Control	TZ	Nano‐CUR	TZ + Nano‐CUR
Initial B.W.	198.3 ± 3.21	201.1 ± 4.11	196.3 ± 2.31	199.6 ± 3.14
Final B.W.	224.1 ± 6.10	189.2 ± 4.33**	223.6 ± 5.22	225.6 ± 2.11
Weight gain	25.8 ± 1.51	−11.9 ± 0.21**	27.3 ± 1.52	26.0 ± 1.21
Liver weight	5.10 ± 0.001	5.30 ± 0.021**	5.00 ± 0.050	5.20 ± 0.031
Relative liver weight	0.0227 ± 0.0005	0.0281 ± 0.0001**	0.0224 ± 0.0005	0.0230 ± 0.0004
Kidney weight	1.56 ± 0.01	1.61 ± 0.3**	1.54 ± 0.07	1.58 ± 0.009
Relative kidney weight	0.0069 ± 0.0001	0.0085±**0.0001	0.0068 ± 0.0009	0.0070 ± 0.0005

Note

Each value is the mean ± SD, *n* = 8. Values marked with ** differ significantly from control value: *p* < .05.

### 3.2 Liver function tests

Oral administrations of TZ for 50 days to adult male albino rats caused a significant increase (*p* < .01) in liver function enzymes’ (AST, ALT, GGT, and ALP) activities, which were (97.32 ± 7.82, 35.51 ± 3.56, 18.54 ± 3.20, and 147.83 ± 9.65 U/L) compared to their control values (48.57 ± 1.75, 11.57 ± 1.75, 5.96 ± 2.23, and 89.62 ± 3.26 U/L), respectively. Whereas, daily oral administrations of Nano‐CUR or mixture of TZ and Nano‐CUR for 50 days did not significantly change the enzyme activities relative to their controls, Table 2. Total bilirubin was significantly (*p* > .01) increased by 95.9% relative to control value due to oral administration of TZ to adult male rats for 50 days. While, the ingestion of Nano‐CUR or TZ + Nano‐CUR mixture showed no significant changes in serum total bilirubin compared to the nontreated Control Group 1, Table 2.

**TABLE 2:** Effect of Tartrazine (TZ) and Nano‐curcumin (Nano‐CUR) on liver functions (enzymes and total bilirubin) in male albino rats

Groups→ Parameters↓	Control	TZ	Nano‐CUR	TZ +Nano‐CUR
AST (U/L)	48.57 ± 1.75	97.32 ± 7.82**	47.83 ± 2.81	49.22 ± 3.60
ALT (U/L)	11.57 ± 1.75	35.51 ± 3.56**	10.12 ± 1.96	12.70 ± 1.90
GGT(IU/L)	5.96 ± 2.23	18.54 ± 3.20**	6.74 ± 2.1	7.53 ± 1.70
ALP (U/L)	89.62 ± 3.26	147.83 ± 9.65**	91.25 ± 6.81	93.10 ± 9.17
T. BiLL (mg/dl)	1.2 ± 0.14	1.9 ± 0,15**	1.3 ± 0.21	1.4 ± 0.22

Note

Each value is the mean ± SE, *n* = 8. Values marked with ** differ significantly from control value: *p* < .01.

### 3.3 Serum blood glucose (sGlu), protein Kinase‐C (PKC), and alpha‐fetoprotein (AFP)

As revealed in Table 3, the level of serum blood glucose (sGlu) was meaningfully augmented by 15.8% in rats ingested with TZ compared to the control value. Whereas, protein Kinase‐C concentration was significantly (*p* < .01) raised in rats treated with TZ (3.98 ± 0.42**Pmol/L) compared to control G1 (0.96 ± 0.31 Pmol/L). On the same respect, AFP concentration was significantly (*p* < .01) increased (102.51 ± 3.53**ng/ml) compared to control (25.27 ± 1.33 ng/ml). While, Nano‐CUR or (TZ + Nano‐CUR mix.)‐ingested rats (G3 and G4 resp.) did not show any significant deviations in PKC or AFP levels related to their control values.

**TABLE 3:** Effect of Tartrazine (TZ) and Nano‐curcumin (Nano‐CUR) on serum blood glucose (sGlu), protein Kinase‐C (PKC), and alpha‐fetoprotein (AFP) in adult male albino rats

Groups→ Parameters↓	Control G1	TZ G2	Nano‐CUR G3	TZ + Nano‐CUR G4
sGlu, mg/dl	169.8 ± 4.23	196.66 ± 4.66**	170.16 ± 3.86	171.87 ± 3.78
PKC, Pmol/L	0.96 ± 0.31	3.98 ± 0.42**	0.95 ± 0.51	4.43 ± 0.61
AFP, ng/ml	25.27 ± 1.33	102.51 ± 3.53**	24.87 ± 2.11	26.56 ± 3.12

Note

Each value is the mean ± SD, *n* = 8. Values marked with ** differ significantly from control value: *p* < .01.

### 3.4 Kidney function and total protein

Ingestion of TZ at a dose of 7.5 mg/kg B.W./day to rats for 50 days significantly (*p* > .01) increased the concentrations of serum urea, creatinine, and uric acid by 252.8%, 100%, and 259.3%, respectively, relative to control. Also, the concentrations of total protein (TP), Alb, Glu, and Alb/Glu ratio were increased by 14.7%, 31.4%, 7.3%, and 21.83%, respectively, relative to control, Table 4. Whereas, there were no significant changes in serum urea, creatinine, uric acid as well as TP, Alb, Glu, and Alb/Glu ratio due to Nano‐CUR (1g/kg B.W./day) or blind of Nano‐CUR (2.0 g) and TZ (7.5 mg)/kg B.W./day ingested to rats for 50 days, Table 4.

**TABLE 4:** Effect of Tartrazine (TZ) and Nano‐curcumin (Nano‐CUR) on kidney functions (urea, creatinine, uric acid, total protein (TP), albumin (Alb), globulin (Glu), and Alb/Glu ratio) in male albino rats

Groups→ Parameters↓	Control	TZ	Nano‐CUR	TZ + Nano‐CUR
Urea	44.41 ± 1.61	156.69 ± 6.42**	45.12 ± 1.35	47.13 ± 1.22
Creatinine	0.51 ± 0.02	1.02 ± 0.07**	0.49 ± 0.04	0.53 ± 0.07
Uric acid	0.43 ± 0.03	1.7 ± 0.06**	0.41 ± 0.5	0.51 ± 0.08
TP (g/dl)	7.62 ± 0.47	8.74 ± 0.83**	7.50 ± 0.43	7.53 ± 1.43
Alb. (g/dl)	4.11 ± 1.04	5.40 ± 1.25*	4.40 ± 2.11	4.46 ± 0.84
Glob. (g/dl)	2.90 ± 2.31	3.13 ± 0.21**	3.01 ± 3.12	3.00 ± 0.99
Alb/Glob. ratio	1.42 ± 1.67	1.73 ± 0.77**	1.50 ± 2.66	1.51 ± 0.92

Note

Each value is the mean ± SE, *n* = 8. Values marked with ** differ significantly from control value: *p* < .01.

### 3.5 Lipid profile

The concentrations of serum total lipids (TL), triglyceride (TG), total cholesterol (TC), high‐density lipoprotein‐C (HDL‐C), low‐density lipoprotein‐C (LDL‐C), and VLDL‐C (very‐low‐density lipoprotein‐C) in rats consumed with TZ (7.5 mg/kg B.W./day) for 50 days were 359.39 ± 5.66, 103.81 ± 6.26, 99.52 ± 10.41, 25.69 ± 2.32, 62.75 ± 5.92, and 12.34 ± 1.36 mg/dl, respectively, which were highly significant (*p* < .01) than those of corresponding controls. The controls were 139.33 ± 2.48, 61.15 ± 2.35, 70.68 ± 1.25, 34.90 ± 2.01, 25.39 ± 2.65, and 10.51 ± 2.21 mg/dl, respectively, Table 5. On the other hand, Nano‐CUR ingested to rats (2.0 g/kg. B.W./day) for 50 days showed no substantial variations in these parameters. However, ingestion of Nano‐CUR and TZ mixture normalized the quantity of these parameters. The percent decreases in TL, TG, TC, LDL‐C, and VLDL‐C due to co‐ingestion of Nano‐CUR with TZ to rats (G4) were 57.9%, 35.7%, 28.0%, 58.4%, and 9.6%, respectively, compared to TZ‐ ingested rats, whereas HDL‐C was increased by 29.2%.

**TABLE 5:** Effect of Tartrazine (TZ) and Nano‐curcumin (Nano‐CUR) on serum lipid profile in rats

Groups→ Parameters↓	Control	TZ	Nano‐CUR	TZ +Nano‐CUR
TL (mg/dL)	139.33 ± 2.48	359.39 ± 5.66**	143.42 ± 32	151.46 ± 53
TG (mg/dL)	61.15 ± 2.35	103.81 ± 6.26**	65.36 ± 2.50	66.76 ± 4.43
TC (mg/dL)	70.68 ± 1.25	99.52 ± 10.41**	66.73 ± 3.93	71.66 ± 5.16
HDL‐C (mg/dL)	34.90 ± 2.01	25.69 ± 2.32**	35.23 ± 3.18	33.18 ± 3.60
LDL‐C (mg/dL)	25.39 ± 2.65	62.75 ± 5.92**	22.82 ± 2.11	26.11 ± 4.63
VLDL‐C (mg/dL)	10.51 ± 2.21	12.34 ± 1.36*	10.73 ± 2.23	11.15 ± 2.27

Note

Each value is the mean ± SE, *n* = 8. Values marked with ** differ significantly from control value: *p* < .01, those marked with *differ significantly from control group: *p* < .05.

### 3.6 Histopathological findings

#### 3.6.1 Liver histology

**FIGURE 2:**
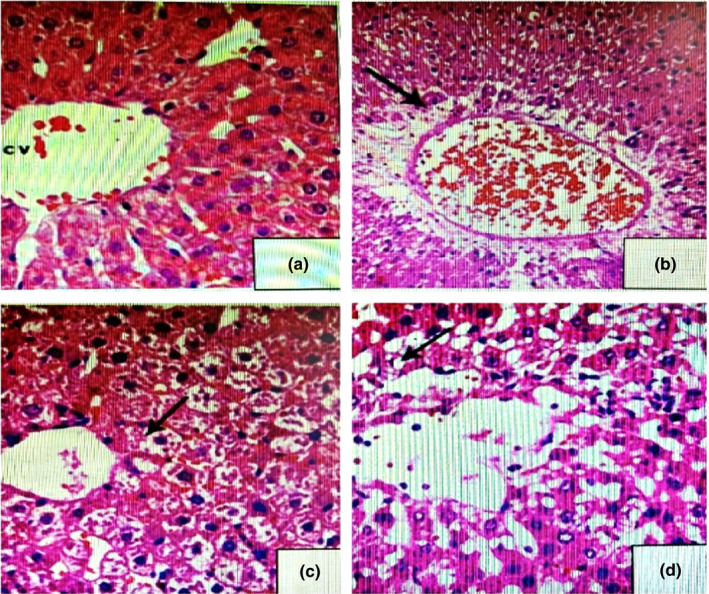
Histopathology of livers of rats ingested Tartrazine (TZ) and the ameliorative effects of Nano‐curcumin (Nano‐CUR). (a) Normal structure of the liver tissue of G1 displaying the normal arrangement of hepatic cords, central vein, normal blood sinusoids(s), and hepatocytes, HE, ×400; (b) Liver tissue of rats ingested 7.5 mg of TZ/kg B.W., displaying dilatation of blood sinusoids and central vein with necrosis and hemorrhage (*), HE, ×400; (c) Liver tissue of rats ingested 2.0 g of Nano‐CUR/kg B.W., displaying a normal arrangement of normal blood sinusoids(s), hepatocytes, and hepatic cords as compared to the controls, HE, ×400; (d) Liver tissue of rats ingested 7.5 mg of TZ + 2.0 g of Nano‐CUR/kg B.W., showing little necrosis (N), hematoxylin and eosin (H&E), ×400

In control rats (Figure 2a), the histopathological examination of liver showed no pathological alterations. Liver tissues of rats showing normal structure, normal hepatocytes with normal nuclei, and normal blood sinusoids appeared between the liver cords. However, liver tissues of animals treated with (7.5 mg/kg B.W.) TZ (Figure 2b) showed vacuolization of hepatocytes and congested blood sinusoids in between the liver cords with intense mononuclear inflammatory cellular infiltration. Liver cells appeared swollen and areas of hemorrhage were noticed between the cells. Nuclei of the cells became flattened‐shaped. The liver tissues of animals treated with 2.0 g of Nano‐CUR/kg B.W. (Figure 2c) showed normal structure, normal hepatocytes with normal nuclei, and normal blood sinusoids as in control. Number of rats displaying histopathological changes in liver was decreased in, (Figure 2d), which had received a mixture of Nano‐CUR with TZ, and the rats in this group revealed only mild necrotic changes and moderate degenerative changes in the hepatocytes.

#### 3.6.2 Kidney histology

**FIGURE 3:**
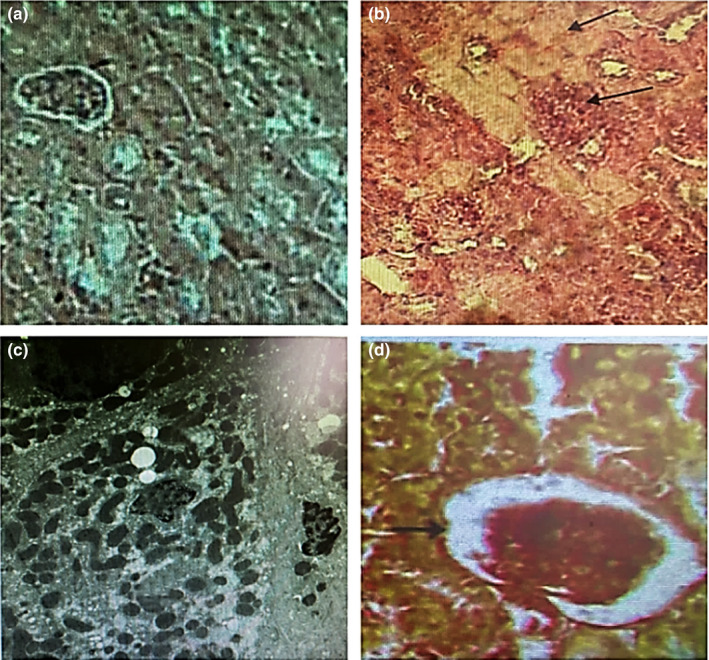
Histopathology of kidneys of rats ingested Tartrazine (TZ) and the ameliorative effects of Nano‐curcumin (Nano‐CUR). (a): Sectional part of G1 rats displays a normal histological appearance of the kidney. (b): Section of the rat kidney treated with 7.5 mg of TZ/(kg B.W.) discovered tubular dilatation with tubular degeneration, thickened basement membrane, and the glomerular capillaries’ dilatation (black arrow). (c): Section of the rat kidney ingested 2.00 g of Nano‐CUR/(kg B.W.) showed near‐normal architecture as compared to control rats. (d): Section of rat kidney ingested a mix. of (7.5 mg of TZ + 2.00 g of Nano‐CUR per kg B.W.) showed slight tubular degeneration and the glomerular capillaries’ dilatation (black arrow)

The histological illustration shows kidneys from each rat group, namely the G1, Figure 3a, the group that administered TZ, Figure 3b, the group that administered Nano‐CUR, Figure 3c, and the group that administered TZ + Nano‐CUR mixture, Figure 3d. In Figure 3a, the kidneys of the rats remained in the normal size, whereas Figure 3b reveals necrosis, tubular degeneration, tubular dilatation with thickened basement membrane, and the glomerular capillaries’ dilatation. Groups that received Nano‐CUR, Fig. (3C), or a blend of Nano‐CUR + TZ, Figure 3d, showed renal histology similar to the structures of the control group, Figure 3a. The two groups show no significant differences from the normal group. Nano‐CUR can reduce or prevent necrosis in epithelial cells of TZ‐treated rats (Figure 3d) significantly (*p* < .05) as compared with TZ‐treated rats, Figure 3b.

## 4 DISCUSSION

### 4.1 Significance of TZ‐ and Nano‐CUR‐ingestion in body, liver, and kidney weights of rats

Relative liver masses and body mass are good indicators of pathology and toxicity (Ezeuko Vitalis et al., 2007). The TZ‐ingested rats showed remarkable declines in body weight gain, whereas the relative liver and kidney weights were found to be increased compared to control (G1). The TZ‐ingestion might lessen the palatability of food or else result in avoidance.

Additionally, TZ‐ingestion might produce the free radicals, which might result in oxidative stress causing body weight losses and metabolic disorders (El‐Desoky et al., 2020a). The reduced weight gain or weight loss and increased liver/kidney weight indicate the sign of toxicity (Ezeuko Vitalis et al., 2007). The increasing liver and kidney weights in TZ‐treated rats may be due to inflammation and enlargement of these organs. On the same respect, Nematbakhsh et al. suggested that the ratio of kidney weight can be used to determine the toxicity, where kidney weight will increase in proportion to the kidney toxicity (Nematbakhsh et al., 2013). They also mentioned that, there was a diminution in capability to concentrate urine and also in papillary hypertonicity in rats exposed to a toxic substance, leading to kidney toxicity. The kidney toxicity causes an inflammation and expansion of tubular cells in the kidneys when exposed to TZ (Figure 3b). The increase in renal weight relates to an increase in serum creatinine and urea levels (Nematbakhsh et al., 2013).

The TZ dye leads to a decrease in the intestinal bacteria population and hinders the food absorption capacity of the intestinal surface causing growth retardation. The azo‐dye reduced to various aromatic amines (sulfanilic acid) in the presence of azo reductase, which are highly carcinogenic and hindered the digestion of food (Chung et al., 1992). Mixing Nano‐CUR with TZ and ingested to rats lead to the increment in the antioxidant capabilities or free radicals scavenging property improving the metabolism and digestion of food. As it is reported that the relative liver weight is involved in judging the pathological disorder of the liver (Hassan et al., 2014). Therefore, an upsurge of weight gains in G4 rats designated the advance effects of Nano‐CUR on various organs of the body, Table 1.

### 4.2 The influences of TZ‐ and Nano‐CUR‐ingestion on liver functions

In the present study, TZ‐administered rats showed the higher activities of hepatic serum enzymes (AST, ALT, GGT, and ALP), T. BiLL., and TP, suggesting injuries, elevated permeability, and hepatic cells’ impairment. Such phenomena help to determine the type and degree of cells’ destruction of the liver. Zhao et al. mentioned that the serum AST, ALT, ALP, and GGT activities are specific for hepatic function and their increase is correlated with the hepatic injury (Zhao et al., 2014). Both ALT and AST activities (located in cytoplasm and mitochondria, respectively) show the injuries of both the mitochondrial and hepatic cellular membranes in TZ‐ingested rats (Rajagopal et al., 2003). Also, Al‐Rejaie et al. mentioned that, the increase of ALT and AST serum levels is specific to hepatocellular disturbance (Al‐Rejaie et al., 2009). The ALP is a liver function enzyme that is related to the membrane lipid in canalicular ducts. ALP increase in serum reflects the biliary flow disturbance. So, the extra‐ or intra‐hepatic interference with the bile flow leads to an elevation of ALP serum levels (Zhao et al., 2014). GGT is a membrane‐bound enzyme, which is present mainly in the canalicular ducts. GGT serum levels are altered by many pathological and physiological factors, such as carcinogenesis and development (Trinder, 1969). The increase of serum GGT level in TZ‐ingested rats may be ascribed to its deliverance from the cellular membrane into blood, which results in the damage to the cellular membrane. The level of serum bilirubin may be raised due to cholestatic injury, acute hepatocellular injury, or biliary obstructions (Mehedi et al., 2013).

Histopathological inspection (Figure 2a) showed swollen hepatocytes, severe hepatic variations, a single large vacuole overcoming the whole cytoplasm, and wide trabeculae from hepatic cells compressing and constricting the sinusoids lumen, and deposition of brown pigment inside the Küpffer cells. The cellular damages resulting beach out of numerous enzymes including ALT, GGT, AS, and ALP into the serum. The higher activities of aminotransferases and augmented serum T. BiLL and TP with the histopathological deviations confirmed each other and recommended that the liver tissue damage was mainly associated with TZ‐ingested rats. Similar results were reported by Amin et al. and Mekkawy et al., indicating that too low or too high doses of artificial dyes contain both carmoisine and TZ which significantly elevated the serum AST, ALT, and alkaline phosphatase activities due to the toxic nature of such dyes (Amin et al., 2010; Mekkawy et al., 1998). Usually, the occurrence of alkaline phosphatase in the sinusoidal and canalicular membranes damages the liver, resulting in a higher serum ALP activity (Kashanian et al., 2012). The significant increases in the aminotransferase level are related to the pathological and biochemical states of the hepatic lobules and letdown to accomplish vital functions of the organs triggering imbalance in intermediary metabolism. Meyer et al., (2017) reported that initial systemic ingestion of TZ in mice indicates a raised serum alkaline phosphatase activity, per portal recruitment of seditious cells and mild per portal liver cell fibrosis.

Administration of Nano‐CUR to rats (2.0 g/kg B.W.) for 50 days (G4) had no changes in hepatic enzyme activities (ALT, AST, GGT, and ALP) or serum T. BiLL or TP concentrations compared to control; this suggests that Nano‐CUR could be safe on liver. On the same respect, administration of blind (TZ + Nano‐CUR) to rats showed significant decreases in the activities of these enzymes when compared with the TZ‐treated group. The concentration of these enzymes approximately came back close to the normal activities. The decreased activities of the hepatic enzymes in the Nano‐CUR‐treated group (G3) support the protective and therapeutic effects of Nano‐CUR. Nano‐CUR is shown to prevent the leakage of hepatic enzymes into the serum and thus supports the hepatocytes of TZ‐induced hepatotoxicity. The substantial reduction in the activities of these enzymes in the Nano‐CUR‐ingested rats indicates a chelating effect of Nano‐CUR and similar results were supported by the histopathological findings of the liver hepatic tissue (Figure 2c).

### 4.3 The Effect of TZ‐ and Nano‐CUR ingested to rats on sGlu, AFP, and PKC

The daily ingestion of TZ to rats for 50 days displayed a substantial upsurge in sGlu concentration when compared to control rats. Similar results were registered by Tawfek et al., (2015) where the sGlu concentration in TZ‐treated rats was (133.89 ± 5.67 mg/dl) compared to the control group (88.03 ± 2.01 mg/dl). Himri, et al. also reported similar findings for the daily intake of TZ for 90 days when compared to control rats (Himri et al., 2012). Whereas, Aboel‐Zahab et al., (1997) observed no significant increases in the sGlu level in rats whose diets were supplemented with varying concentrations of TZ and carmoisine.

The elevation of glucose level in TZ‐ingested rats may be due to stimulation of gluconeogenesis and glycogenolysis by the liver with a temporary loss of endocrine functions, and the peroxidation of membrane lipids which promotes the creation of thiobarbituric acid species reacts with cytochrome P450 (Al‐Shinnawy & Elkattan, 2013; Baynes, 1991; Pfeifer & McCay, 1971). The individual Nano‐CUR‐ingestion (G3) showed no obvious (*p* < .05) variations in the sGlu level compared to control (G1) rats, indicating the safety effect of Nano‐CUR on the metabolism of glucose. The combined ingestion of Nano‐CUR with TZ (G4) suppresses the sGlu level in the TZ‐treated rats (G2) to be the same as G1 rats. The chelating and therapeutic effect of Nano‐CUR against TZ‐induced hepatotoxicity improves glucose metabolism and regulates the blood level. A similar research was performed by Zhang, et al. who confirm the significant role of curcumin in diabetes and its associated disorder treatments, since it favorably affects insulin resistance, hyperlipidemia, hyperglycemia, and necrosis and islet apoptosis (Zhang et al., 2013). Also, it prevents the harmful complications of diabetes, instead of its poor bioavailability and limited solubility. Therefore, in the current experiment, we have prepared Nano‐CUR, which is highly soluble in aqueous solution, highly bioavailable, and has tremendous benefits with respect to the health improvement.

Our study revealed that TZ administration to the adult male albino rats significantly (*p* < .01) increased PKC and AFP levels, indicating the adverse effects of TZ on different metabolism and body systems. The reason for such activities can be explained as: TZ has been considered as a crucial regulatory component for maintaining various cellular activities including gene growth, expression, exocytosis, and differentiation. Much evidence indicates the direct activation of PKC isoforms due to the generation of ROS depending on the cell type, specific isoform and the ROS generation sites can be involved in either cell damage/death or protection via hyperglycemia. Hyperglycemia culminates in an overproduction of oxygen and Sorbitol producing from the enzymatic conversion of glucose and as a result decreases the formation of antioxidant reducing equivalents. The role of PKC has been found to promote the creation of endogenous ROS to induce a positive feedback loop. Hence, under pathological environments a general signaling mechanism is prompted in mitochondrial dysfunction and the redox state of the cell becomes imbalanced. It may phosphorylate potent transcription activator and increase the oncogene expression, which can promote cancer progression (Daniela et al., 2012). Several hyperglycemia‐induced mechanisms may create vascular dysfunctions including increased polyol pathway flux, altered cellular redox state, increased production of diacylglycerol, the subsequent activation of PKC isoforms, and formation of various glycated end‐products. The ingestion of TZ + Nano‐CUR mixture normalizes the PKC activities, confirming the therapeutic and protective effects of Nano‐CUR against TZ‐induced diabetic nephropathy. Curcumin produces both PKC‐α and PKC‐β1 blockers, which are potential for adjuvant therapy and used for the treatment of diabetic nephropathy.

Alpha‐fetoprotein (AFP) is a fetal‐specific glycoprotein that is produced in the developing fetus and found in adults. The high levels of AFP in the blood of an individual may be a sign of liver failure, damage, or liver cancer (Blohm et al., 1998). The results of the current research backing the harshness of TZ on rat liver, as significantly increased serum AFP in the TZ‐induced rats, were noticed compared to control, G1. Whereas, Nano‐CUR administration to rats causes no changes in serum AFP indicating its safety in liver. Ingestion of Nano‐CUR + TZ to rats has an ameliorative effect on the AFP content compared with its level in TZ‐treated rats. These results were in accordance with Ahmed et al., (2014), who mentioned that the AFP serum level had been elevated in diethyl nitrosamine (DENA)‐treated rats and CUR treatment has an ameliorative effect on DENA (Ahmed et al., 2014; Onyema et al., 2006).

### 4.4 Influence of TZ and nano‐CUR on kidney functions

The research outcomes demonstrate that the daily TZ‐intake significantly upsurges serum creatinine, urea, and uric acid levels, while it decreases the serum albumin compared to the control rats. The principal end‐product of protein catabolism is blood urea, which is a noble indicator for functioning kidney. The production of urea in the liver happens through the urea or ornithine cycle. The blood urea is excreted through the renal tubules and the urea nitrogen level in the blood is maintained via the excretory function of the kidneys. The increase of urea nitrogen level in the TZ‐ingested rats takes place owing to a decrease in renal activity and the reabsorption. Urea is normally reabsorbed in the proximal and distal tubule, where only in the proximal tubule almost 40%–50% of the filtered urea is reabsorbed (Ecelbarger et al., 2001). The blood urea increased in all types of kidney dysfunction where the deposition of calcium takes place as hypervitaminosis D (Varley, 1954). Creatinine (Mol. Wt., 113 Da) appears in the serum proportional to the body's muscle mass and its rising level leads to a poor performance of kidneys (Inuwa et al., 2011). Thus, there is a link between TZ‐ingestion and renal impairment, including tubular reabsorption, glomerular filtration rate, and renal blood flow. Usually, creatinine is excreted through kidneys, while the filtration ability of kidney decreases during kidney dysfunction and hence it increases the serum creatinine. A twofold increase of serum creatinine decreases 50% kidney function and a threefold increment declines the kidney function by 75% (Sandhiutami et al., 2019).

It has been reported that the substantial raise of urea and creatinine leads to the impairment of renal function (Mehedi et al., 2013; Timbrell, 2009). Furthermore, the declined ability of the kidney to filter fluid within the body leads to increase serum urea and creatinine. However, TZ‐ingestion increases the functional capacity of tubular excretion which interfered with creatinine metabolism (Manal Said & Nawal, 2012).

Albumin measures the nephrotoxicity of TZ and the decreased albumin levels were recorded in the present research, indicating the toxic effect of the TZ dye on renal and hepatic tissues. Usually, TZ can cause necrosis of podocyte cells, which play an important role in albumin filtration in the glomerulus. Necrosis of podocyte cells causes larger pore formations in the glomerulus and hence albumin (proteins with large molecular weights) can pass during the glomerular filtration causing the decrease in serum albumin level (Kumar et al., 2017).

Moreover, TZ exposure to rats may produce an oxidative stress induced adverse influence on the renal function. Figure (3B) shows that TZ‐ingestion in rats damages the glomeruli through inflammatory mechanism, kidney apoptosis, dilatation of the glomerular capillaries, and atrophy of renal corpuscles. These results demonstrate how it amplified the glomerulus and podocytes’ cell permeability, and consequently decreased the albumin level in the blood. These results are in parallel with Himri et al.’s findings for the rats group ingested with TZ (Himri et al., 2011).

Curcumin has a low bioavailability due to its lower solubility in water, extensive first cross metabolism, and low intestinal permeability (Siviero et al., 2015). However, the formation of Nano‐CUR increases the curcumin bioavailability and the ingestion of Nano‐CUR to rats (2.0 g/kg B.W.) for a 50‐day time duration, and no significant variations in kidney functions were noticed, suggesting that Nano‐CUR could be safe on the kidney. On the same respect, ingestion of TZ + Nano‐CUR mixture to rats for the same time significantly reduces the serum concentrations of urea, uric acid, and creatinine, while the albumin was increased compared to the TZ‐treated rats. The decreased concentrations of the aforementioned parameters in Nano‐CUR‐treated rats indicate its defensive and therapeutic effects on kidneys. Nano‐CUR‐ingestion removes the toxic substance from the blood by increasing the secretory function of kidney and thus helps to protect the kidney tissues from TZ‐induced nephrotoxicity. Additionally, Nano‐CUR cotreated group increases the Reno protector activity via chelating effect. The histopathological findings showed that Nano‐CUR‐ingestion improves the kidney tissues, as shown in (Figure 3c).

### 4.5 Influence of TZ on serum lipid profiles

The significant upsurges in serum TL, TG, TC, HDL‐C, LDL‐C, and VLDL‐C concentrations obtained in this work are in proper agreement with those of Aboel‐Zahab et al., who supplemented rats with varying concentrations of TZ and carmoisine (Aboel‐Zahab et al., 1997). Himri et al. indicated the same results as well for TZ‐ingested rats with significant increases in the cholesterol and triglyceride levels compared to control (Himri et al., 2011). Tawfek et al. reported that rats received 20 mg/kg B.W. TZ/daily for 60 days, which considerably augmented the levels of TG, TC, LDL‐C, and HDL‐C compared to the control group (Tawfek et al., 2015). Ashour and Abdelaziz results were different from the obtained outcomes of the current research, who reported a significant decline in serum TG and TC levels due to oral ingestion of fast green to male albino rats for 35 days (Ashour & Abdelaziz, 2009). In addition, the significant decrease in HDL‐cholesterol for TZ‐ingested rats was noticed, owing to imbalances between the normal rates of secretion and fat metabolism. The significance of TZ‐ingestion in lipid profile and increasing cholesterol level is observed, owing to the membrane structure and function disruption, which directly influence its permeability, fluidity, transport system, and activity of associated enzymes (Mohamed et al., 2015). However, the ingestion of Nano‐CUR (G3) or Nano‐CUR with TZ (G4) successfully normalized the TL, TG, TC, HDL‐C, LDL‐C, and VLDL‐C levels with respect to G1. The substantial diminutions in lipid and lipoprotein concentrations in Nano‐CUR‐ingested rats may be attributed to the significant escalation in cholesterol catabolism by the activity of cholesterol‐7a‐hydroxylase enzyme, which converts cholesterol to bile acids and eliminates it from the body (Babu & Srinivasan, 1997). Also, Akira mentioned that curcumin's influence on various lipoproteins (LDL‐C, HDL‐C, and VLDL‐C) resembles the drugs—cholestyramine, mevastatin, lovastatin, and simvastatin that are used for correcting the imbalance in serum lipoproteins in patients with diabetes and coronary heart disease (Akira & Kishimoto, 1992). These drugs are known to increase HDL‐C and decrease LDL‐C. Suresh Babu and Srinivasan mentioned that the cholesterol decrease caused by dietary curcumin in the diabetic state was exclusively from the LDL and VLDL fractions of the lipoprotein (33% decrease). HDL‐C was increased by 25% in diabetic animals (Babu & Srinivasan, 1997). Furthermore, they found significant decreases in blood triglyceride and phospholipids (40% and 24%, respectively) were brought about in diabetic animals maintained on curcumin diet. The decreases in TG, TL, TC, and VLDL‐C and LDL‐C lipoproteins and increases in HDL‐C levels due to Nano‐CUR‐ingestion were found to be more prominent than the results reported by Suresh Babu and Srinivasan (Babu & Srinivasan, 1997). This observation may be due to the conversion of CUR to Nano‐CUR, which increases its solubility and bioavailability in the animal system.

## 5 CONCLUSION

The influences of Nano‐CUR administration against TZ‐induced changes in liver, kidney histology, and related functions were investigated. TZ‐ingestion significantly increased sGlu, AFP, PKC, as well as lipid profiles, whereas there was a significant (*p* < .01) decrease in HDL‐C and albumin concentrations compared to control. TZ‐ingestion caused histopathological changes in liver including dilatation of blood sinusoids, and central vein with hemorrhage and necrosis. Remarkably, the Nano‐CUR‐ingestion protected the rat's kidney and liver of TZ‐ingested rats, which are evidenced by a substantial reduction in all kidney functions, liver function enzymes, and lipid profile. The significant improvement in serum albumin and HDL‐C levels was also recorded. The normal blood sinusoids(s), normal arrangement of hepatic cords, and hepatocytes in liver tissues were obtained in Nano‐CUR‐ingested rats compared to the controls. The same results were found in the section of rat kidney showing near‐normal architecture as compared to control rats. The liver tissue of rats ingested with Nano‐CUR showed little necrosis. In addition, the section of rat kidney ingested a mix. of (7.5 mg of TZ + 2.00 g of Nano‐CUR/kg B.W.) showed mild tubular degeneration. Similarly, Nano‐CUR corrects the imbalance in serum glucose, AFP, PKC, and lipid profiles in TZ‐ingested rats compared to control.

## CONFLICTS OF INTEREST

The authors declare no conflict of interest.

## AUTHOR CONTRIBUTIONS

**Gaber E. El‐Desoky:** supervision (equal). **Mohamed A. Habila:** methodology (equal).
